# Quantitative Modeling of Climate Change Impacts on Mycotoxins in Cereals: A Review

**DOI:** 10.3390/toxins13040276

**Published:** 2021-04-12

**Authors:** Cheng Liu, H. J. Van der Fels-Klerx

**Affiliations:** Wageningen Food Safety Research, 6708 WB Wageningen, The Netherlands; cheng.liu@wur.nl

**Keywords:** deoxynivalenol, fusarium toxins, aflatoxins, adaptation, food safety, food safety management, wheat, maize, rice

## Abstract

Our climate is projected to change gradually over time. Mycotoxin occurrence in cereal grains is both directly and indirectly related to local weather and to climate changes. Direct routes are via the effects of precipitation, relative humidity, and temperatures on both fungal infection of the grain and mycotoxin formation. Indirect routes are via the effects of the wind dispersal of spores, insect attacks, and shifts in cereal grain phenology. This review aimed to investigate available modeling studies for climate change impacts on mycotoxins in cereal grains, and to identify how they can be used to safeguard food safety with future climate change. Using a systematic review approach, in total, 53 relevant papers from the period of 2005–2020 were retrieved. Only six of them focused on quantitative modeling of climate change impacts on mycotoxins, all in pre-harvest cereal grains. Although regional differences exist, the model results generally show an increase in mycotoxins in a changing climate. The models do not give an indication on how to adapt to climate change impacts. If available models were linked with land use and crop models, scenario analyses could be used for analyzing adaptation strategies to avoid high mycotoxin presence in cereal grains and to safeguard the safety of our feed and food.

## 1. Introduction

Mycotoxins are a chemically diverse group of low molecular weight contaminants that may have toxic effects on animal and human health. They are secondary metabolites produced by specific fungi, mainly *Aspergillus* spp., *Fusarium* spp., and *Penicillium* spp., upon and after infection of crops by these fungi, and under conducive conditions related to fungal infection and toxin production. Mycotoxins are of great concern worldwide because of (i) their abundant presence in major feed and food commodities, such as cereal grains, (ii) high chemical stability during feed and food processing, and (iii) their possible negative effects on animal and human health.

In Europe, legislative limits have been set for the maximal presence of mycotoxins in feed and food and their commodities, with the aim to protect animal and human health. Such maximum limits have been set for the presence of deoxynivalenol (DON), nivalenol (NIV), T-2 toxin, HT-2 toxin, and zearalenone (ZEN), as well as for aflatoxins in food and food commodities via Commission Regulation (EC) 1881/2006. Further, maximum limits have been set for the presence of aflatoxin B_1_ at 20 µg/kg in all feed materials, via Directive 2002/32/EC, mainly because of the transfer of this mycotoxin to milk-producing animals via feed, resulting in the excretion of aflatoxin M_1_ in milk. For some other mycotoxins, including ZEN, DON, ochratoxin A, and fumonisins B_1_ and B_2_, guidance values have been set for their maximal presence in cereal and cereal products, except for maize by-products, via Commission Recommendation 2006/567/EC.

Climate change impacts are expected to generally increase the occurrence of mycotoxins in our feed and food commodities [1,2]. Although the impact of climate change differs per region (see Section 2.1), the anticipated increasing rainfall and higher temperature in some regions may lead to more conducive climatic conditions for *Fusarium* spp. in Europe. The expected more frequent and prolonged periods of drought may stimulate aflatoxin production by *Aspergillus flavus* in both pre-harvest and post-harvest conditions [3,4,5].

To be able to deal with the increased mycotoxin occurrence in feed and food commodities in the future, it is necessary to estimate the anticipated effects, i.e., to quantify the future impacts of climate change effects on mycotoxin presence in cereal grains in various regions around the world. To this end, forecasting models for the presence of toxin-producing fungi and related mycotoxins in cereal grains can be used. Such models can be fed with data on climate change effects on the climate variables, notably temperature, rainfall, and relative humidity (RH), and the resulting prediction of toxin levels in crops can be investigated on the regional (e.g., province) level.

This review aimed to investigate the available models on climate change impacts on mycotoxins in cereal grains, and how they can be used to safeguard food safety with future climate change. First, we summarize the changes in climate variables and their impacts on mycotoxin production (Section 2). Second, we present available quantitative modeling studies on climate change impacts on mycotoxins in cereals grains resulting from a literature review (Section 3 and Section 4). Third, we discuss similarities and differences between the modeling studies, the available evidence, and provide directions on how to use the models to safeguard food safety in the future (Section 5). Finally, the method used in this literature review is presented in Section 6. This study is not focused on comparing different models at a technical level, but rather reviews their contributions to climate change impact studies as a whole.

## 2. Climate Change Impacts

Climate is commonly defined as the weather patterns averaged over a long time. The standard averaging period is thirty years. Studying the impact of climate change, therefore, requires the comparison of climate over several decades. Climate scenario analysis is the common practice to answer the “what if” question. It is important for food safety researchers to realize the difference between weather and climate to really analyze the impacts of climate change on food safety, in terms of temporal duration and projection uncertainties.

### 2.1. Changes in Climate Variables

Climate change is a worldwide challenge, especially for agriculture. Changes in temperature, distribution of precipitation (including more extreme events, such as floods and droughts), and moisture content have already been observed [6]. The prevailing line of thought within the scientific community states that these changes are caused by an increase in the radiation balance of the Earth due to greenhouse gas emissions. Climate models are run for different scenarios to determine projected changes in climate variables worldwide. These changes differ by scenario and region, but generic changes are as follows:

a. Temperature has increased worldwide since the start of observations in 1654. The temperature is expected to rise at a rate of 0.03 °C/year [7]. Modeling studies project that temperature will continue to increase gradually over time, resulting in a 2–5 °C increase of the 1-in-20 year extreme daily maximum temperature by the end of the 21st century [8]. The highest temperature increases will be over land and at high northern latitudes. In Southern Europe, changes may result in an increase of 4–5 °C with longer drought periods, resulting in decreasing crop yields. In areas of Western and Atlantic Europe, changes of 2.5–3.5 °C with drier and hotter summers are expected. In Central Europe, an increase of 3–4 °C, higher rainfall, and floods are forecasted to negatively affect crop harvest, although longer growing periods may benefit crop yields. Northern Europe would expect an average temperature increase of 3–4.5 °C, with a significant increase in precipitation of 30–40%. This will lead to increases in crop yields and new crop cultivation patterns [5,9].

b. Total precipitation is expected to increase in some regions (e.g., high latitude and tropical regions, and in winter in the northern mid-latitudes) and to decrease in others (e.g., Southern Europe and the Mediterranean regions, Central Europe, central North America, Central America and Mexico, northeast Brazil, and southern Africa) [8]. The distribution of precipitation is also expected to change, resulting in an increase in the number of extreme precipitation events. These events might intensify floods or droughts in some agricultural areas and seasons [6].

c. The atmosphere and soil moisture are affected by evapotranspiration changes, due to expected temperature and precipitation changes. These changes lead to a higher moisture content of the atmosphere at a rate of about 7% for every 1 °C rise [10]. The annual mean soil moisture decreases in the Mediterranean region and subtropics, and increases in East Africa, Central Asia and some other regions with increased precipitation [6]. These direct changes in climate variables further influence crop development, fungal infection, and mycotoxin formation.

### 2.2. Climate Change Impacts on Mycotoxins (Qualitative Studies)

**Figure 1:**
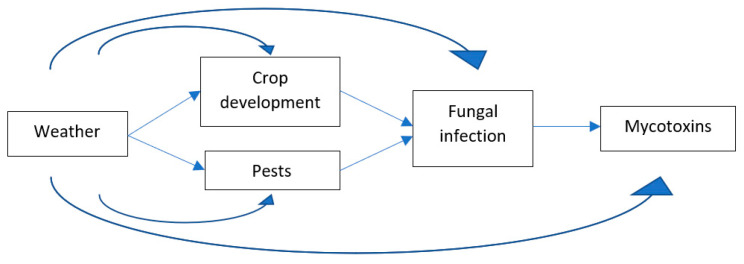
Direct and indirect effects of weather on mycotoxins in cereal grains.

Local weather and changes thereof have direct and indirect effects on the presence of mycotoxins in agricultural crops (Figure 1). First, weather—in combination with other factors such as the crop variety—affects the development of crops. The most important is the effect of temperature on the crop flowering, ripening, and full maturation dates. The most critical stage for fungal infection of cereals grains is the period around flowering. The United Kingdom (UK) Climate Impacts Program projected weather would advance the date of onset of wheat anthesis by approximately two weeks by 2050 and maturity for harvest by three weeks [11]. Therefore, if the weather is inducive around grain flowering, the probability of fungal infection is highest.

Weather also has a direct effect on fungal infections. Wind and rain splash will increase the distribution of spores. Climate change is expected to favor the growth, virulence, multiplication, persistence, and range expansion of the most serious wheat and maize pests. The Food and Agriculture Organization (FAO) has recently published a report [2] on climate change and food safety, specifically discussing climate change impacts on pest damage and mycotoxins. Insects can carry fungal spores, which are introduced into the plants when the insects feed. Plants that are stressed by pest damage are more predisposed to fungal infections. These changes are opening new geographic areas for disease outbreaks and insect attacks, which increase the chances of fungal infection and mycotoxin formation [12,13].

Lastly, the production of mycotoxins by fungal species upon/after grain infection is influenced by weather conditions. Drought stress increases the production of aflatoxins by *Aspergillus flavus* in maize, and humid and warm conditions favor the production of DON by Fusarium species in wheat.

Increased climate variability is expected to increase the probability of mycotoxin accumulation not only in fields, but also after harvest, including in commercial and traditional storage facilities [2,7]. The risk of aflatoxin and ochratoxin production in food may increase as a result of inadequate storage and transport conditions across changing climate zones. Pests in silos could multiply more rapidly under elevated temperature, producing more metabolic water. Condensation and wet pockets can initiate mold formation with the possibility for an increase in contamination with mycotoxins, such as ochratoxin A, aflatoxins, and trichothecenes, in damp grain [7].

## 3. Quantitative Modeling Climate Change Impacts on Fusarium Toxins

Using a literature review focusing on research articles that report on modeling climate change impacts on mycotoxins in cereal grains, only six of such quantitative studies were retrieved, of which three focused on Fusarium toxins.

Madgwick et al. [14] developed a weather-based logistic regression model using a wheat growth model as input to project the incidence of Fusarium head blight in the UK under five climate scenarios: baseline (1960–1990), high CO_2_ concentration in the 2020s, low CO_2_ concentration in the 2020s, high CO_2_ concentration in the 2050s and low CO_2_ concentration in the 2050s (Table 1). The wheat growth model projected the wheat anthesis dates for the 2020s and 2050s under all five climate change scenarios. The wheat growth model suggested that the average temperature in May and precipitation in the second week of June were required as input for the Fusarium head blight model. Hence, the input data of Madgwick et al.’s model are “May average temperature” and “precipitation in the second week of June”. The output of the model is the percentage of plants affected by Fusarium head blight in the region within 80 km of Rothamsted, UK. Although this exercise focused on Fusarium head blight rather than mycotoxins, the method is able to be extended to fusarium toxins. Wheat anthesis dates were projected to get progressively earlier with climate change by about 11–15 days across the country, possibly caused by high CO_2_ concentration. The incidence of Fusarium head blight was related to rainfall during anthesis and temperature during the preceding six weeks [14]. The Fusarium head blight epidemics were also projected to be more severe, especially in southern England by the 2050s.

Van der Fels-Klerx et al. [15,16] used an empirical modeling approach to estimate climate change impacts on DON in wheat in northwestern Europe by 2040 (Table 1). In addition to direct effects of climate change on mycotoxin contamination as input, the authors also considered the indirect effects related to a change in wheat flowering and full maturation date. Existing empirical models for wheat phenology and DON prediction for northwestern Europe [15] were used and fed in with gridded synthetic weather data for each of the three scenarios (baseline, and two future scenarios). A baseline scenario (1975–1994) was used, as well as two future scenarios (moderate and extreme) for the period of 2031–2050. The future scenarios used two different combinations of general circulation models (GCMs) and regional climate models (RCMs) for climate change projections, which were MPI/KNMI (RACMO2) and METOHC/METOHC (HADRM3Q0). The output of the model is the DON concentrations under different scenarios in Norway, Sweden, Finland, and the Netherlands at a 50 km by 50 km spatial scale. Climate change projection data were extracted from ENSEMBLES and downscaled using the LARS weather generator. Both flowering and full maturation dates were projected to be one to two weeks earlier in the cultivation season due to climate change effects. DON concentrations were generally projected to be increased—in some cases up to three times the concentration in the baseline period—depending on the scenario and the region (grid) considered.

Joo et al. [17] developed a censored regression model for estimating the impacts of climate change on ZEN contamination of rice grains in South Korea (Table 1). The model used temperature and relative humidity during flowering and harvest as the main meteorological variables, together with rice variety (brown or white) and region. They used this regression model to compare the ZEN contamination in the current situation, the 2030s, and the 2050s under representative concentration pathway (RCP) emission scenarios RCP 2.6, RCP 4.5, and RCP8.5. Their model results are in line with other quantitative studies on relationships between temperature and mycotoxin presence: the average temperature has a positive relationship with the production of mycotoxins until the harvest date. The climate scenario analysis in this study projected that ZEN contamination in rice grains will increase in South Korea in the 2030s and 2050s, particularly on the western side of the country, due to the higher temperature during harvest in those areas.

## 4. Quantitative Modeling Climate Change Impacts on Aflatoxins

Three of the six quantitative modeling studies focused on aflatoxins, all in pre-harvest maize. Chauhan et al. [18] developed a mechanistic model to predict aflatoxins in maize (Table 1). This model uses ambient temperature, radiation, rainfall, soil water, and soil nitrogen to simulate maize growth and yield on an area basis with daily time steps. The model performed well in simulating the climatic risk of aflatoxin contamination in maize, as indicated by a significant positive correlation between the aflatoxin risk index (ARI) and the measured aflatoxin B1 in crop samples, which was 0.69 for a range of rainfed Australian locations and 0.62 when irrigated locations were also included in the analysis. Rather than using future climate data, Chauhan et al. studied the probabilities of ARI exceeding a given value using 106 years of historical climatic data. Different agronomic management scenarios were applied to understand the role of management practice in aflatoxin risk control. As a result, dry and hot climates had a combined effect that triggered a higher probability of higher ARI compared with having either dry or hot conditions alone. Scenario analysis on management practices suggested that under non-irrigated conditions, the risk of aflatoxin contamination could be minimized by adjusting sowing time or selecting an appropriate cultivar for lower temperature and water stress conditions. Chauhan et al. concluded that the grain filling period is crucial for agronomic practices to reduce the severity of drought or exposure of the crop to high temperatures, in order to minimize the risk of aflatoxin contamination. One limitation of this model is the inability to account for insect damage in the computation of ARI.

Battilani et al. performed a study that aimed to estimate aflatoxin contamination in pre-harvest maize and wheat in Europe with future climate change (Table 1). A mechanistic model, “AFLA-maize”, was used to predict aflatoxin contamination risks. A weather simulator was used to obtain 100 years of stochastically generated daily weather data for each scenario covering 2254 grids (50 × 50 km) in Europe. Daily gridded weather data were then used as inputs into the crop (maize, wheat) phenology and the aflatoxin prediction model (AFLA-maize). The maize phenology model estimated the future maize flowering and harvest dates, which were also used as input into the AFLA-maize model. The model was further run under +2 °C (Scenario +2 °C, 2030–2059) and +5 °C (Scenario +5°, 2070–2099) scenarios relative to a baseline period (1975–2005). The output of the model is the aflatoxin cumulative index (AFI) calculated by summing the daily AFIs till maize harvest, for each grid and scenario per simulation run (100 years). An AFI value of ≥95 was considered to correspond to exceedance of the EC legal limit of 5 μg of aflatoxin per kg of raw maize kernels. In the baseline scenario, mean AFI for maize computed over all grids in Europe was 38. Thirty-nine percent of grids in Europe had a mean AFI calculated above 0 and 20% of the grids were predicted to have an AFI above the current legal limit in Europe. In the +2 °C and the +5 °C scenarios, the mean AFIs were 73 and 95, respectively. Mean AFI increased by 92% and 149% when moving from the baseline scenario to the +2 °C and +5 °C scenarios, respectively, and 39% and 54% of the sites had an AFI > 95.

In their study, an AFLA-wheat model was developed, based on the principles of AFLA-maize. In the baseline scenario, the mean AFI for wheat was 0.7, and *A. flavus* growth (AFI > 0) was estimated to be possible in more than 90% of the sites, though with very low AFI values. In the +2 and +5 °C scenarios, mean AFIs increased by 60% and more than doubled, respectively, but were still relatively low, implying that aflatoxin contamination in wheat with future climate change is not very likely.

Van der Fels-Klerx et al. [20] estimated the impact of climate change on aflatoxin B_1_ in maize and aflatoxin M_1_ in milk, using a model chain approach (Table 1). This approach was applied to the case study of maize grown in Eastern Europe and used as dairy cows’ compound feed in the Netherlands [23,24]. The models used in this study included: a preliminary version of the forecasting model for aflatoxin B_1_ in maize (a combination of mechanistic modeling and a Bayesian network), named PREMA [21], and a carry-over model of aflatoxin B_1_ in maize to aflatoxin M_1_ in milk [22]. The data(bases) used included climate change data, maize phenology data, data on the composition of compound feed for dairy cows (inclusion rates of ingredients used for compound feed formulation), and a database with the results of national control programs for mycotoxins in feed ingredients. The flowering and harvest dates of maize were estimated using the Joint Research Centre (JRC) weather databases Agri4cast and the crop phenology model WOFOST [25]. These JRC weather data and estimated maize flowering and harvest dates were used as input into the aflatoxin forecasting model which simulates the dispersal and growth of *Aspergillus flavus* and aflatoxin B_1_ production in maize. The model was then run under the baseline condition (2005–2017) and for IPCC A1B scenarios in 2030 using projection results from three different climate models: DMI, ETHZ, and METO. Finally, aflatoxin B_1_ contamination was estimated for 10,000 iterations, and the mean and standard deviation for the predicted aflatoxin B_1_ in maize grown in Eastern Europe were calculated on a grid basis. The overall mean of the aflatoxin B_1_ concentration, over all iterations and all grids, was 0.8 μg/kg for the baseline situation. For the future 2030 climate, the overall mean was estimated to decrease with the DMI climate model (to 0.6 μg/kg) but to increase with the ETHZ (to 1.2 μg/kg) and METO (to 1.6 μg/kg) models. Regional differences were observed, but with all three climate models, aflatoxin B_1_ levels were expected to increase in Eastern Europe.

## 5. Discussion and Conclusions

This review investigated the available quantitative modeling studies on climate change impacts on mycotoxins in cereal grains, as the basis for an evaluation on how these models can be used to safeguard food safety with future climate change. Only six of such quantitative modeling studies were available, all focusing on the pre-harvest stage of cereal grain production. Three studies focused on Fusarium toxins and three studies focused on aflatoxins.

These quantitative studies and their results have some similarities. All six studies used climate change scenario analysis or long-term climate data to analyze the impacts of climate change. This is needed to differentiate weather, climate, and climate change, and more importantly to include the spatial and temporal variability of local and regional climate in the consideration. All studies used fungal/mycotoxin prediction models with a range of weather variables, which provide easy handles for climate change scenario analysis. Most studies also used crop phenology input into the fungal/mycotoxins models to account for indirect effects of climate change impacts on crop phenology. Regarding crop phenology, those studies report similar findings in that climate change will most likely shift grain flowering time by one to two weeks earlier in most regions. Results from the fungal/mycotoxin models show that—in general—mycotoxin contamination in cereal grains will increase with climate change, but regional differences are expected, with some regions expecting lower mycotoxin levels. All these model studies focused on the impact assessment of climate change effects on food safety. A one-way linear relationship was used in this process (Figure 1).

As can be seen from the study descriptions, the six studies obviously used different climate change scenarios, different cereal grain types and mycotoxins, and focused on different geographical areas. Basically, the six studies focused on either DON/ZEN in wheat and rice, or on aflatoxins in maize. The cereals oats, barley, and sorghum are less intensively studied. These cereals are, however, also cultivated in smaller amounts globally as compared to wheat and maize. Other type of mycotoxins, such as fumonisins, have not been studied in the context of climate change impact quantification. The model types used were either empirical and/or mechanistic approaches. However, despite the differences in the six studies, the general results were quite similar in that all studies show an expected overall increase in mycotoxins in cereals grains with climate change. In some regions, however, the future climate conditions are no longer expected to be conducive for fungal infection and/or mycotoxin production. This is related to the interaction of the changes in the flowering of the grain and the climate in that critical grain cultivation period with climate change. The conditions in which there is less fungal infection and/or mycotoxin production in future climate scenarios are interesting for further investigation, since they can bring some insight into possible management strategies to adapt to climate change, for instance, by choosing a cereal grain variety with either a later or earlier flowering date, or choosing a completely different cereal grain type in certain, vulnerable regions.

The next step that is thus urgently needed is to use the models to give directions for *adaptation* to climate change to assure mycotoxin levels in grains stay below legal limits for raw materials and derived feed and food, and to safeguard feed and food safety. Mycotoxin forecasting models can be used for short-term adaptation by running different scenarios for the input variables related to agronomics in the field. Such what-if scenarios could be run, for instance, for using a different grain variety which is more resistant to fungal infection or which will have a different flowering date. By investigating the predicted toxin levels of the models, the effects of such agronomics could be studied, and used to give advice to farmers. Additionally, these models can be used to give directions for long-term adaptation to climate change effects. If the models predict the mycotoxin presence to be extremely high in the future in a certain region, for instance, then it can be decided to grow alternative crops in that region. If mycotoxin forecasting models were linked to land use models (e.g., the iCLUE model [26]) and crop phenology models, such scenarios could be investigated, and used to give directions on the use of our land and cultivation of crops in such a way that safe feed and food can be produced under the estimated climate change effects.

To conclude, this review showed that forecasting models for mycotoxins in cereal grains have been used to estimate climate change impacts on mycotoxin presence, often in combination with crop phenology models to also account for climate change effects on the cereal grains. However, only six of such quantitative modeling studies using climate change scenarios are available, focusing on Fusarium toxins and aflatoxins in the pre-harvest stage. The climate change scenario analysis clearly showed an expected overall increase of mycotoxins in cereals grains with future climate scenarios, though regional differences are observed, with some regions even having lower expected mycotoxin levels in future. The available studies do not show how to adapt to climate change. Further climate change scenario analysis is highly recommended for use in quantitative studies to understand the regional differences of climate change impacts on mycotoxin production in cereal grains to investigate ways to adapt to climate change.

## 6. Material and Methods

**Figure 2:**
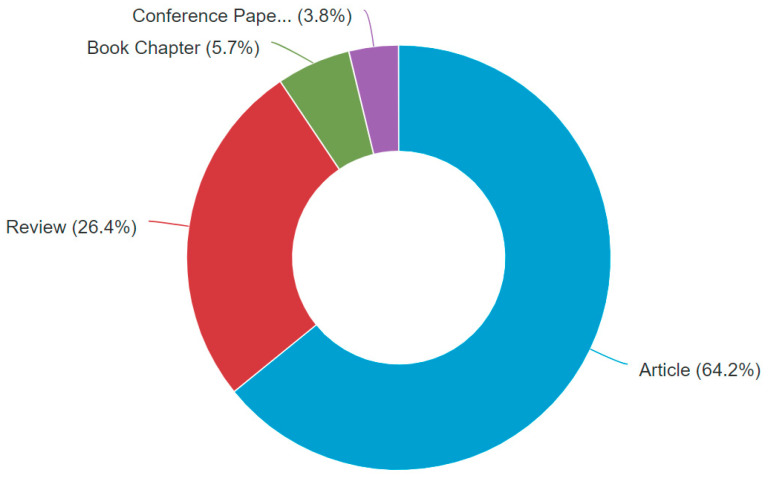
Document type of the selected 53 search results related to modeling climate change impacts on mycotoxins in maize and cereals in Scopus (accessed date: 14/12/2020).

We used the database Scopus and search strings (model* AND climate* AND mycotoxin*) between 2005 and 2020. Up to December 2020, in total, 53 papers were retrieved that were related to cereal grains, such as wheat, maize, oats, barley, and rice. These 53 search results were further analyzed and reviewed in this study. Figure 2 indicates that 64.2% of the search results are articles and more than one quarter (14) of the retrieved articles are review papers. Among all 53 papers, the UK has published the most (13) on this topic, followed by the Netherlands (9), Italy (8), and Germany (8). The quantitative studies are 45% of the search results. Only six publications were research studies on modeling the impacts of climate change on mycotoxins in agricultural crops using scenario analysis. All six studies focused on the cultivation period of cereals till harvest, of which two studies focused on wheat, three on maize, and one on rice.

## Figures and Tables

**Table 1:** Overview of models for quantitatively studying the impact of climate change on mycotoxins.

Model	Type of Model	Scenarios Used	Input	Output	Crop	Mycotoxin/FHB	Country/Region	Reference
Madgwick et al.	Logistic regression, wheat growth model	Five scenarios: Baseline (1960–1990), low and high CO_2_ concentrations in 2020s and 2050s	Average temperature in May, precipitation in the second week of June	Percentage of plants affected by Fusarium head blight	Wheat	Fusarium head blight incidence	Region within 80km of Rothamsted, UK	[14]
Van der Fels-Klerx et al.	Multi-variable regression, crop phenology model	Three scenarios: Baseline (1975–1994), moderate and extreme scenario from 2031–2050	Temperature, precipitation, relative humidity, wheat flowering, and full maturation date	DON ^1^ concentration at harvest	Wheat	DON	Northwestern Europe	[15,16]
Joo et al.	Censored regression model	Baseline, RCP2.6, RCP4.5, and RCP8.5 in the 2030s and 2050s	Temperature and relative humidity at flowering and temperature at harvest, region, type of rice	ZEN ^2^ concentration in rice grains at harvest	Rice	ZEN	South Korea	[17]
Chauhan et al.	Simulation model	Management scenarios to deal with dry and hot climates	Temperature, radiation, rainfall, soil water and soil nitrogen, yield	Aflatoxin risk index	Maize	Aflatoxin	Australia	[18]
Battilani et al.	Mechanistic model	Baseline: 1975–2005, +2 °C (2030–2059), +5 °C (2070–2099)	Temperature, relative humidity, precipitation, leaf wetness, water activity, flowering and harvest dates	Aflatoxin cumulative index	Maize and wheat	Aflatoxin	Europe	[19]
Van der Fels-Klerx et al.	Model chain approach including a combination of mechanistic and Bayesian network model, and a simulation model	Baseline: (2005–2017), IPCC ^3^ A1B in 2030	Temperature, precipitation, relative humidity, wind speed, flowering and harvest dates, compound feed composition	Aflatoxin B1 concentration at harvest and aflatoxin M1 in milk	Maize	Aflatoxin	Eastern Europe	[20,21,22]

^1^ deoxynivalenol, ^2^ zearalenone, ^3^ Intergovernmental Panel on Climate Change.

## Data Availability

Not applicable.
